# Assessment of cardiovascular event reduction with SGLT2 inhibitors compared to GLP-1 receptor agonists in type 2 diabetes mellitus

**DOI:** 10.6026/973206300213000

**Published:** 2025-09-30

**Authors:** Vijaya Krishna Prasad Vudathaneni, Swetha Bharathi Nadella, Kalikrishna Varaprasad Movva, Phanindra Dulipala, Ramanarayana Boyapati

**Affiliations:** 1 Department of Internal Medicine, Columbus Regional Hospital, 2400 17thSt, Columbus, Indiana, United States of America; 2 Department of Anaesthesiology, Anaesthetics, Principal House Officer, Mackay Base Hospital, Mackay, Queensland, Australia; 3 Department of Community Medicine, Katuri Medical College and Hospital, Guntur, Andhra Pradesh, India; 4 Department of Periodontology, Sibar Institute of Dental Sciences, Takkellapadu, Guntur, Andhra Pradesh, India

**Keywords:** Type 2 diabetes mellitus, SGLT2 inhibitors, GLP-1 receptor agonists, cardiovascular events, MACE, heart failure

## Abstract

Cardiovascular disease (CVD) remains the leading cause of morbidity and mortality in individuals with type 2 diabetes mellitus (T2DM). Antidiabetic agents, sodium-glucose co-transporter 2 inhibitors (SGLT2i) and glucagon-like peptide-1 receptor agonists (GLP-1 RA) have demonstrated cardiovascular benefits, though direct comparative data are limited. This retrospective cohort study analysed records of 300 T2DM patients treated between January 2021 and December 2023 at a tertiary care centre. Patients were divided into two groups: Group A (n=150) received SGLT2i (empagliflozin or dapagliflozin), and Group B (n=150) received GLP-1 RA (liraglutide or semaglutide). Major adverse cardiovascular events (MACE), including myocardial infarction, stroke, and cardiovascular death, were tracked over 24 months. Group A showed a lower incidence of MACE (11.3%) compared to Group B (15.3%), though the difference was not statistically significant (p=0.182). Kaplan-Meier curves indicated slightly better event-free survival in the SGLT2i group. Haemoglobin A1c reduction was similar between the groups (1.2% vs. 1.3%, p=0.456), while hospitalization for heart failure was significantly lower in the SGLT2i group (6.0% vs. 10.6%, p=0.048). Thus, we show that both SGLT2 inhibitors and GLP-1 receptor agonists offer cardiovascular protection in T2DM, with SGLT2i providing a modest advantage in reducing MACE and a significant benefit in lowering heart failure hospitalizations.

## Background:

Type 2 diabetes mellitus (T2DM) is a progressive metabolic disorder characterized by insulin resistance, impaired insulin secretion, and chronic hyperglycemia. It is a major global public health concern, with increasing prevalence across both developed and developing nations. According to recent estimates, more than 500 million individuals worldwide are affected by diabetes, with T2DM accounting for over 90% of all cases [1]. One of the most critical complications of T2DM is the heightened risk of cardiovascular disease (CVD), which remains the leading cause of mortality in this population [2, 3]. Cardiovascular events such as myocardial infarction, stroke, and heart failure occur more frequently in diabetic patients than in the general population, necessitating therapeutic strategies that address both glycemic control and cardiovascular risk [4]. In recent years, antidiabetic pharmacotherapy has undergone significant evolution with the emergence of novel drug classes such as sodium-glucose co-transporter 2 inhibitors (SGLT2i) and glucagon-like peptide-1 receptor agonists (GLP-1 RA). These agents have shown promising outcomes in reducing not only blood glucose levels but also cardiovascular morbidity and mortality in high-risk patients [5, 6]. SGLT2 inhibitors, including empagliflozin and dapagliflozin, act by preventing glucose reabsorption in the renal proximal tubules, thereby promoting glycosuria and modest weight loss. Beyond glycemic benefits, clinical trials have demonstrated their ability to significantly lower the risk of heart failure hospitalization and cardiovascular death [7, 8]. On the other hand, GLP-1 receptor agonists such as liraglutide and semaglutide exert their glucose-lowering effects through enhancement of glucose-dependent insulin secretion, delayed gastric emptying, and appetite suppression. More importantly, they have been associated with a reduction in atherosclerotic events, including myocardial infarction and stroke, in multiple cardiovascular outcome trials [9, 10]. Despite the cardioprotective properties of both drug classes, there is an ongoing debate regarding which class offers superior cardiovascular protection, especially in patients without established CVD. While previous studies have individually assessed the cardiovascular benefits of SGLT2 inhibitors and GLP-1 receptor agonists, head-to-head comparisons remain scarce in clinical literature. Furthermore, patient-specific factors such as age, comorbidities, and baseline cardiovascular risk may influence therapeutic outcomes and guide personalized treatment selection. Therefore, the present study aims to compare the efficacy of SGLT2 inhibitors versus GLP-1 receptor agonists in reducing major adverse cardiovascular events (MACE) among patients with T2DM in a real-world clinical setting.

## Materials and Methods:

This retrospective cohort study was conducted at a tertiary care hospital to evaluate and compare the cardiovascular outcomes associated with the use of sodium-glucose co-transporter 2 (SGLT2) inhibitors and glucagon-like peptide-1 receptor agonists (GLP-1 RA) in patients with type 2 diabetes mellitus (T2DM). Ethical approval for the study was obtained from the institutional ethics committee prior to data collection.

## Study population:

Medical records of adult patients aged 40-75 years with a confirmed diagnosis of T2DM and no prior history of cardiovascular events in the preceding 6 months were reviewed. Inclusion criteria were patients who had been prescribed either SGLT2 inhibitors (empagliflozin or dapagliflozin) or GLP-1 receptor agonists (liraglutide or semaglutide) for a minimum period of 6 months. Patients with chronic kidney disease stage 4 or 5, hepatic impairment, or active malignancy were excluded from the study.

## Study design and grouping:

A total of 300 eligible patient records from January 2021 to December 2023 were included and divided into two groups:

Group A (SGLT2i group): 150 patients receiving empagliflozin or dapagliflozin.

Group B (GLP-1 RA group): 150 patients receiving liraglutide or semaglutide.

## Data collection:

Demographic details, clinical history, duration of diabetes, comorbidities, baseline HbA1c levels, body mass index (BMI), blood pressure, lipid profile, and medication history were recorded. Cardiovascular outcomes, including myocardial infarction, stroke, cardiovascular-related death, and hospitalization due to heart failure, were documented over a follow-up period of 24 months. Data were obtained through electronic health records and physician documentation.

## Outcome measures:

The primary outcome was the incidence of major adverse cardiovascular events (MACE), defined as a composite of non-fatal myocardial infarction, non-fatal stroke, and cardiovascular death. Secondary outcomes included hospitalization for heart failure and changes in HbA1c levels from baseline to the end of the follow-up period.

## Statistical analysis:

Data analysis was carried out using SPSS software version 25.0 (IBM Corp., Armonk, NY, USA). Continuous variables were expressed as mean ± standard deviation (SD) and compared using the independent t-test. Categorical variables were presented as frequencies and percentages and analyzed using the Chi-square test. Event-free survival was assessed using Kaplan-Meier curves and compared using the log-rank test. A p-value of less than 0.05 was considered statistically significant.

## Results:

A total of 300 patients with type 2 diabetes mellitus were included in the study, with 150 patients in each treatment group. Both groups were comparable at baseline with respect to age, gender distribution, BMI, HbA1c levels, and the prevalence of hypertension and dyslipidemia. The mean age of participants in the SGLT2 inhibitor group (Group A) was 59.2 ± 8.4 years, while in the GLP-1 receptor agonist group (Group B), it was 58.7 ± 7.9 years (p = 0.621). Both groups had a nearly equal gender distribution (Group A: 52% males; Group B: 50% males). The mean baseline HbA1c was 8.4 ± 0.9% in Group A and 8.5 ± 1.0% in Group B (p = 0.432). No significant differences were observed in baseline characteristics (Table 1 - see PDF). Over the 24-month follow-up period, the incidence of major adverse cardiovascular events (MACE) was lower in the SGLT2i group (11.3%) compared to the GLP-1 RA group (15.3%), though the difference was not statistically significant (p = 0.287). However, the rate of hospitalization due to heart failure was significantly lower in Group A (6.0%) than in Group B (10.6%) (p = 0.048), indicating a favorable effect of SGLT2 inhibitors on heart failure prevention (Table 2 - see PDF). Both groups demonstrated significant reductions in HbA1c levels from baseline. Group A showed a mean HbA1c reduction of 1.2%, while Group B showed a reduction of 1.3% at 24 months. The difference between groups was not statistically significant (p = 0.456), suggesting similar glycemic control efficacy (Table 3 - see PDF). The Kaplan-Meier survival analysis showed a trend toward better cardiovascular event-free survival in the SGLT2i group, although this difference was not statistically significant (log-rank p = 0.193) (data not shown in tables). Overall, SGLT2 inhibitors demonstrated a slightly superior cardiovascular profile, particularly in reducing heart failure-related hospitalizations, without compromising glycemic control (Tables 1-3 - see PDF).

## Discussion:

The present study aimed to compare the cardiovascular outcomes of two prominent classes of antidiabetic medications-SGLT2 inhibitors and GLP-1 receptor agonists-in patients with type 2 diabetes mellitus (T2DM). While both classes have been shown to significantly reduce cardiovascular risk in various randomized controlled trials, direct comparative evidence in real-world clinical settings remains limited. Our findings suggest that SGLT2 inhibitors may offer a marginally superior protective effect against major adverse cardiovascular events (MACE), particularly in reducing heart failure-related hospitalizations, while both drug classes demonstrated comparable efficacy in glycemic control. Consistent with our results, previous landmark trials such as EMPA-REG OUTCOME and DAPA-HF have demonstrated the cardioprotective effects of SGLT2 inhibitors in reducing hospitalization for heart failure and cardiovascular mortality, even in non-diabetic populations [1, 2]. Empagliflozin, in particular, reduced cardiovascular death by 38% and heart failure hospitalizations by 35% in high-risk T2DM patients [1]. Similarly, the DAPA-HF trial reported a 26% relative risk reduction in worsening heart failure or cardiovascular death with dapagliflozin [2]. Our study found a significantly lower rate of heart failure admissions in the SGLT2i group (6.0% vs 10.6%), reinforcing these observations. GLP-1 receptor agonists, on the other hand, primarily reduce atherosclerotic cardiovascular events rather than heart failure outcomes. This was demonstrated in the LEADER and REWIND trials, where liraglutide and dulaglutide significantly reduced the incidence of stroke and myocardial infarction in patients with established or at high risk for CVD [3, 4]. Our study did show slightly higher rates of myocardial infarction and stroke in the GLP-1 RA group; however, the differences were not statistically significant. These findings are consistent with the known differential effects of the two drug classes-SGLT2 inhibitors having more pronounced benefits in heart failure and GLP-1 RAs showing benefits in atherosclerotic disease [5, 6] . Both classes of drugs showed comparable glycemic control in our study, with mean HbA1c reductions of 1.2% and 1.3% for SGLT2i and GLP-1 RA, respectively. This is consistent with previous reports indicating that while both classes are effective in reducing HbA1c, their cardiovascular benefits extend beyond glycemic effects [7, 8]. The glucose-independent mechanisms, including diuresis, natriuresis, reduction in arterial stiffness, and anti-inflammatory effects for SGLT2i, and anti-atherogenic, anti-inflammatory, and weight-lowering effects for GLP-1 RAs, are believed to contribute to their cardiometabolic advantages [9, 10]. Patient adherence and tolerability also influence treatment outcomes. SGLT2 inhibitors are generally administered orally and are well-tolerated, though they may be associated with genitourinary infections [11]. GLP-1 RAs, which are injectable agents, can pose a challenge in terms of patient compliance due to gastrointestinal side effects and injection-related discomfort [12]. Such factors, although not measured in our study, may influence the overall effectiveness and real-world applicability of these therapies. It is also important to consider renal outcomes when selecting between these drug classes. SGLT2 inhibitors have shown remarkable benefits in slowing the progression of diabetic kidney disease [13], while GLP-1 RAs also offer renal protection, though to a lesser extent [14]. The renal benefits of SGLT2i may have indirectly contributed to better cardiovascular outcomes, particularly heart failure-related events, as cardiorenal interplay is a recognized mechanism in diabetes management [15]. Despite its strengths, this study has limitations. The retrospective design inherently carries the risk of selection bias and unmeasured confounders. The study was conducted at a single center, which may limit the generalizability of the results. Additionally, the follow-up period of 24 months, although adequate for assessing intermediate outcomes, may not capture long-term cardiovascular benefits. Future multicenter prospective trials are warranted to confirm these findings and explore the comparative long-term benefits of these two drug classes.

## Conclusion:

In conclusion, our study supports the existing evidence that both SGLT2 inhibitors and GLP-1 receptor agonists offer significant cardiovascular benefits in patients with T2DM. While the overall MACE rates were slightly lower in the SGLT2i group, the reduction in heart failure hospitalizations was statistically significant. Treatment selection should be individualized, considering the patient's cardiovascular profile, comorbid conditions and tolerance to therapy.
